# Fully covered stents are similar to semi-covered stents with regard to migration in palliative treatment of malignant strictures of the esophagus and gastric cardia: results of a randomized controlled trial

**DOI:** 10.1007/s00464-017-5441-0

**Published:** 2017-02-24

**Authors:** Jan Persson, Ulrika Smedh, Åse Johnsson, Bo Ohlin, Magnus Sundbom, Magnus Nilsson, Lars Lundell, Berit Sund, Erik Johnsson

**Affiliations:** 1 Department of Surgery, Institute of Clinical Sciences, Sahlgrenska University Hospital, Sahlgrenska Academy at the University of Gothenburg, 41345 Gothenburg, Sweden; 2 Department of Radiology, Institute of Clinical Sciences, Sahlgrenska University Hospital, Sahlgrenska Academy at the University of Gothenburg, Gothenburg, Sweden; 3 Department of Surgery, Blekinge Hospital, Karlskrona, Sweden; 4 Department of Surgical Sciences, Uppsala University, Uppsala, Sweden; 5 Division of Surgery, CLINTEC, Department of Surgical Gastroenterology, Karolinska Institute at Karolinska University Hospital, Huddinge, Stockholm, Sweden

**Keywords:** Stent, Esophagus cancer, Palliation, Dysphagia, Stent migration

## Abstract

**Introduction:**

Stent migration is a significant clinical problem in palliation of malignant strictures in the esophagus and gastro-esophageal junction (GEJ). We have compared a newer design of a fully-covered stent to a widely used semi-covered stent using migration >20 mm as the primary outcome variable. Effects on dysphagia, quality of life (QoL) and re-intervention frequency were also investigated.

**Methods:**

Patients with dysphagia due to non-curable esophagus/GEJ cancer were randomized to receive either a more recent design of a fully-covered stent (n = 48) or a conventional semi-covered stent (n = 47). Chest x-ray, dysphagia and QoL were studied at baseline, one week, four weeks and three months thereafter.

**Results:**

There were no significant differences either in stent migration distance or in the migration frequency. Stent migration during the total study period occurred in 37.2 % in the semi-covered group compared to 20.0 % for the fully-covered group. Dysphagia was measured with Watson and Ogilvie scores and with the dysphagia module in the QoL scale (QLQ-OG25). On average, there was a tendency to better dysphagia relief for the fully-covered design as scored with the two latter dysphagia instruments (p= 0.081 and p= 0.067) at three months and towards more re-interventions in the semi-covered group (p= 0.083).

**Conclusion:**

In spite of its somewhat lower intrinsic radial force, the fully-covered stent was comparable to the conventional semi-covered stent with regard to stent migration. The data further suggest a potential benefit of the fully-covered stent in improving dysphagia in patients with longer life expectancy.

The incidence of cancers of the esophagus and gastro-esophageal junction (GEJ) is rising in the Western world [1]. The majority of patients are not eligible for curative treatment due to either an advanced tumor stage or a poor general condition [2, 3]. A very important goal is thus to provide optimized palliative care and to maintain or improve quality of life (QoL). Dysphagia is the most important symptom to relieve, since it affects QoL most negatively [3, 4]. In recent years endoscopically placed self-expandable metallic stents (SEMS) have become a well-documented, effective and widely used palliative treatment for dysphagia, in particular among patients with a life expectancy of less than 3 months [4–7].

The first commercially available SEMS in the 1990s were uncovered and were associated with a re-intervention rate of up to 30–50%, most commonly due to stent obstruction secondary to tumor or inflammatory tissue in-growth [8, 9]. A variety of different SEMS designs equipped with a plastic lining covering the exterior surface of the stent were subsequently developed to prevent this complication [10]. Unfortunately, whereas the smooth lining of the first generation of covered stents helped to prevent tumor in-growth, those SEMS adhered less well to the esophageal wall. A common and acknowledged disadvantage of fully covered SEMS (fcSEMS) is therefore a high risk (20–39%) for stent dysfunction due to dislocation [11–15] leading to recurrent or insufficient relief of dysphagia. Semi-covered stents (scSEMS), i.e., covered in the mid portion but with bare mesh endings were developed; these cause less migration-related events although re-obstruction in the uncovered endings may still occur [11, 16, 17]. Despite these limitations scSEMS are commonly used throughout the world. When this study was launched, the scSEMS were the most widely used stents for palliation of cancers of the esophagus and cardia in Sweden, in particular the Ultraflex stent, which therefore was chosen to this study. In recent years, newer designs of fcSEMS modified to reduce the risk of migration have been developed. The fcSEMS used in this study (Wallflex^®^) differs from earlier generations of fcSEMS designs in several aspects. It has its silicone lining on the interior luminal side of the stent, which leaves the rough surface of the braided stent mesh facing the mucosa. These modifications, together with a different shape of the flare at the endings, are in theory thought to reduce stent migration. Another important factor to consider when studying stent migration is the inborn radial force of the stent. The fcSEMS Wallflex^®^ has a slightly lower radial force compared to the well-established scSEMS Ultraflex^®^ [18], which theoretically could entail a higher risk for migration. Whether the Wallflex^®^ fcSEMS is comparable to the conventional Ultraflex^®^ scSEMS with regard to migration is not yet known.

Previous studies addressing the advantages or limitations of different stent designs in palliation of cancers of the esophagus and cardia have focused on parameters such as QoL, dysphagia relief or need for endoscopic re-interventions due to perceived symptoms as the main outcome measures. However, there is little evidence available concerning the degree of actual stent migration with regard to stent designs. To our knowledge, earlier investigations have consistently shown higher migration rates of fcSEMS regardless of the design [11–15]. In this study, we therefore tested the hypothesis that the most widely used conventional scSEMS (Ultraflex^®^) is associated with less stent migration compared to a more modern fcSEMS design (Wallflex^®^). The primary endpoint of this prospective, randomized study was to compare these stent designs with regard to migration over time using direct measurements in conventional chest X-ray images. The need for re-interventions, effects on dysphagia and QoL, technical failures and survival were used as secondary endpoints.

## Methods

### Inclusion

Between 2011 and 2014, 95 patients with incurable cancer in the esophagus or the GEJ were asked to participate in this prospective randomized study. The patients were recruited from four university hospitals and two district hospitals. The demographic background characteristics of the groups are shown in Table 1. Before the endoscopy, the patients were randomized to receive either an fcSEMS or a scSEMS through a web-based computer-aided system (DynaReg Generic system). By minimization, the groups were stratified between a distal tumor margin of <3 cm, or >3 cm from the GEJ in order to avoid dissimilarities between the groups with regard to the level of the stricture.

**Table 1:** Background characteristics of the patients included

	scSEMS (n = 47)	fcSEMS (n = 48)	*p* value
Age year median (min–max)	72.2 (48.2–91.0)	71.2 (56.8–91.0)	0.539^1^
Gender (*n* = female)	11 (23.4%)	13 (27.1%)	0.635^2^
Distal margin from cardia <3 cm (*n* = yes)	33 (70.2%)	35 (72.9%)	0.635^2^
Tumor length (cm) median (min–max)	6.0 (1.0–18.0)	6.0 (1.0–14.0)	0.576^1^
Tumor type (*n* = adenocarcinoma)	32 (34.8%)	35 (38.0%)	0.143^2^
Metastatic disease (*n* = yes)	25 (53.2%)	29 (60.4%)	0.367^2^
Dilatation during stent procedure	2 (4.2%)	0 (0.0%)	0.149^2^

Two-tailed Mann–Whitney *U* test^1^ or Pearson Chi-Square test^2^ was used for data evaluation. *p* < 0.05 was considered significant

The *inclusion* criteria were as follows: (1) Biopsy-verified squamous cell carcinoma or adenocarcinoma in the esophagus or the GEJ where stent-treatment is applicable; (2) Age >18 years; (3) swallowing difficulties with a severity of dysphagia of 2–4 according to Ogilvie [19]; (4) Curative treatment not possible; (5) Written informed consent obtained from the patient.

The *exclusion* criteria were as follows: (1) Other concurrent malignancy that might impact the life span and/or QoL of the patient; (2) Inability to understand or complete the written questionnaire; (3) Need for a stent with the upper margin less than 2 cm from the upper esophageal sphincter; (4) Need of more than one stent to bridge the tumor.

### Procedure

**Fig. 1:**
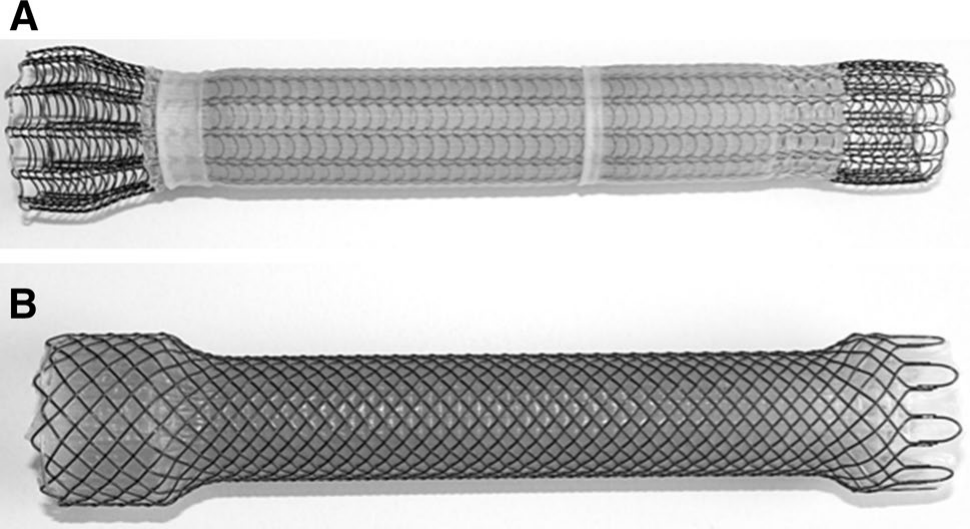
Photograph depicting the stents that were examined in this study with the proximal endings to the left. **A** The semi-covered Ultraflex^®^ stent; **B** the fully covered Wallflex^®^ stent

The patients were randomized to receive either a scSEMS (Ultraflex^®^ Esophageal NG Stent System Boston Scientific) or an fcSEMS (Wallflex^®^ fully covered Esophageal stent Boston Scientific). The patients were not informed about which stent they received. The Ultraflex^®^ stent consists of a knitted nickel-titanium alloy (Nitinol) wire tube and has a polyurethane layer, which covers the midsection of the stent extending to within 1.5 cm of either end of the stent (Fig. 1). The stent used in this study had a proximal flare of 23 mm and an inner body diameter of 18 mm. It was available in three lengths: 100, 120, and 150 mm. The Wallflex^®^ stent is made of a multiple-wired mesh of Nitinol and has a full silicone internal covering with progressive step-flared ends (Fig. 1). The body diameter of the stent used was 18 mm, and the flare diameters were 25 mm proximally, and 23 mm distally. This stent was available in three lengths: 103, 123, and 153 mm. All available stent lengths were used in both groups.

The vast majority of patients undergoing the endoscopic procedure were treated under conscious sedation with midazolam, and alfentanil or pethidine in addition. The upper and when possible also the lower margin of the tumor was marked with a metal clip. If it was impossible to pass the tumor with the endoscope, the length of the obstruction was determined by the radiological findings or by the use of an “on the table” conventional contrast X-ray. Under fluoroscopic X-ray guidance, a guide-wire was passed down to the stomach. When the endoscope had been removed, the stent was inserted over the wire and positioned in relation to the clips. The length of the stent was chosen according to the length of the stricture, and the stent was placed with at least a 2 cm proximal and distal overlap to the upper and lower margins of the tumor. In cases where the stent was positioned with its distal end below the cardia, the distal overlap was aimed at 1 cm. Dilatation was not done routinely to be able to pass the tumor with the endoscope. However, if there were difficulties passing the stricture with the introducer of the stent, a dilatation was performed up to a maximum of 12 mm. Technical failure was defined as inability to place a stent due to technical problems during the initial procedure, or any other event on day 0 that made further participation impossible. Immediately after stent placement, its position was documented with a postero-anterior and lateral chest X-ray. The majority of the patients were examined in the standing position. The patients received additional written and oral information after receiving the stent. These instructions included advice on diet and ingesting only liquid nutrients for the first 3 days after the procedure.

### Follow-up

The follow-up was scheduled at 1 week, 4 weeks, and 3 months after the procedure. A chest X-ray was performed on each occasion and the patients filled in the dysphagia and QoL questionnaires. An independent secondary survey of the patient’s medical records at the treating hospital was done after a patient’s death to ensure that any stent-related events and re-interventions had been recorded.

### Variables

*Migration* was evaluated by measurements on conventional frontal and lateral chest X-rays by an experienced consultant thoracic radiologist. The stent position was measured on the lateral projection in relation to thoracic structures such as the thoracic vertebrae and the aortic arch. All images were stored and analyzed on the same work station (Advantage Workstation VolumeShare 5 (AW 4.6), GE Healthcare, Waukesha, WI, U.S.A. with Multisync LCD 1990SXI NEC monitors) to minimize any procedure-related differences. It was decided that a stent displacement of >20 mm would be defined as a stent migration. This value was chosen in order to avoid over-interpretation of stent movements mainly due to two reasons; first, some foreshortening of a stent due to its expansion occurs and second, since differences in the level of inspiration might affect the location of the reference anatomical structures within the thoracic cavity. Another rationale was that movements less than 20 mm might not be of clinical significance, since each stent was routinely placed with a 20 mm overlap.

Total dislocation was defined as no stent being visible in the esophagus at X-ray or at endoscopy. The total dislocations were included as a stent migration >20 mm event at the subsequent follow-up and were also evaluated separately.

*Re-interventions* the indication for re-interventions was patient complaints of sudden or progressive inability to swallow regardless of X-ray findings.

*Dysphagia* was investigated with two well-known instruments, the Watson dysphagia score [20, 21] and the Ogilvie score [19, 22], as well as with a symptom-oriented quality of life instrument that has a module that captures information regarding swallowing difficulties (QLQ-OG25) [23].

*Health-related quality of life* (QoL) was measured with validated instruments originating from the European Organization for Research and Treatment of Cancer (EORTC), which has a generic instrument that measures global QoL in patients with cancer (QLQ-C30) [24] and a more symptom-specific instrument that is developed for cancer in the esophagus and stomach (QLQ-OG25) [23]. These are well known, validated instruments with the added benefit of having normal reference values for the healthy population [25, 26].

### Statistics

A sample size of 43 patients in each group was calculated based on an estimate [11–15] that the expected rate of migration in the conventional scSEMS group was 10% and in the group with fcSEMS 35%, whereupon a corresponding difference could be detected with a power of 80% and a significance level of 95%, (*p* < 0.05). The SPSS statistical program was applied for data analysis. The point prevalence of data was compared using parametric or non-parametric tests where appropriate. A *p* value of less than 0.05 was considered statistically significant.

### Ethics

This study was performed according to the Declaration of Helsinki and the Ethical Review Act. The study protocol was approved by the Ethical Review Board Authority in Stockholm (protocol 2009/3:7). Written informed consent was obtained from each participant before inclusion in the trial. The trial is registered in the ClinicalTrials.gov (NCT 02166320).

## Results

**Fig. 2:**
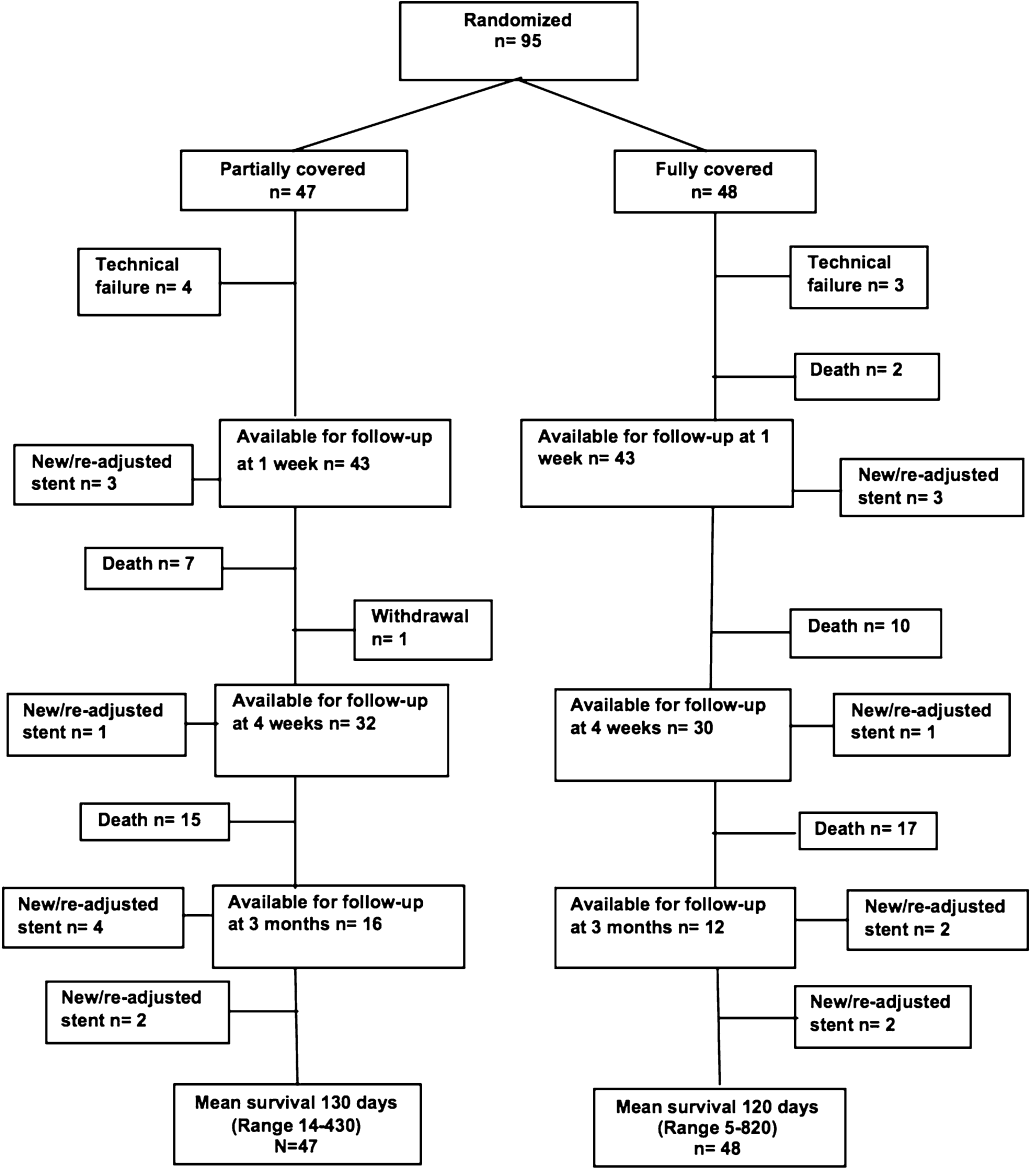
Flow chart showing numbers of patients included in each arm for evaluation of the primary variable, stent migration. Technical failures were defined as either impossibility to place a stent, or any other event that occurred on the day of inclusion that made further evaluation impossible. New/re-adjusted stent was defined as stent migration or dislocation to such an extent that the patient needed a new stent or a re-adjustment of the original one

Ninety-five patients were included, and 86 patients were available for the initial analysis of the primary outcome variable at 1 week. Because of the nature of this aggressive disease, the number of patients in each group diminished for every scheduled follow-up (Fig. 2). At the scheduled follow-up at 1 week, 15 out of 95 randomized patients had left the study, which means that 80 patients were available for the initial analysis of the secondary variables (2 were dead; 7 had had technical failure; 6 stents had migrated, 3 in each group, to an extent that re-intervention was necessary) (Fig. 2). In 2 patients in the fcSEMS group and 3 patients in the scSEMS group, it was impossible to pass the tumor with the guide-wire. Two additional patients were excluded on day 0 (1 scSEMS patient suffered a perforation requiring surgery and 1 fcSEMS patient did not get the allocated stent). These patients were thus denoted as technical failures. There were no differences in demographic background characteristics between the two groups (Table 1).

### Stent migration

Over a period of 3 months, 6 out of 45 patients (13.3%) in the fcSEMS group and 11 out of 43 (25.6%) of the patients in the scSEMS group experienced stent migration. During the total survival span of the patients, stent migration occurred in 9 out of 45 (20.0%) of the patients in the fcSEMS group compared to 16 out of 43 (37.2%) of the patients in the scSEMS group, a difference that was not significant (Table 2). In addition, there was no significant difference between the groups at any time point with regard to the migration distance (Table 2). An interesting observation was that stent migration occurred not only in the distal direction but in the oral direction as well, although less frequently (Table 2). In the scSEMS group, 2 patients experienced total stent dislocation at 1 week. At 4 weeks, no patients, and at 3 months, 1 patient, experienced total dislocation in this group. The corresponding figures in the fcSEMS group were 1, ,1, and 0.

**Table 2:** Stent migration > 20 mm, migration distance in millimeters and numbers of endoscopic re-interventions

	scSEMS	fcSEMS	*p* value
Stent migration >20 mm number of cases			
At 1 week	4	4	0.971^1^
At 1 month	3	1	0.293^1^
At 3 months	4	1	0.154^1^
Total numbers at 3 months	11 (25.6%)	6 (13.3%)	0.145^1^
Total number during survival	16 (37.2%)	9 (20.0%)	0.068^1^
Migration distance in millimeters			
1 week median (min–max)	2.0 (−20 to 70)	0.0 (−30 to 144)	0.243^2^
1 month median (min–max)	5.5 (−39 to 50)	2.5 (−16 to 91)	0.415^2^
3 months median (min–max)	10.5 (−11 to 42)	0.0 (−7 to 33)	0.270^2^
Proximal migration at one week (*n* = yes)	8 (24.2%)	13 (36.1%)	0.231^1^
Numbers of endoscopic re-interventions			
1 week	6 (13.9%)	5 (11.6%)	0.744^1^
1 month	5 (15.6%)	2 (6.7%)	0.288^1^
3 months	4 (25.0%)	1 (8.3%)	0.322^1^
Total interventions at 3 months	15 (34.9%)	8 (18.6%)	0.083^1^

Measured at 1 week, 4 weeks, and 3 months after stent placement. Migration in proximal direction is denoted as negative values. The accumulated numbers at 3 months and during the subjects’ life span are also shown

Pearson Chi-Square test^1^ or two-tailed Mann–Whitney *U* test^2^ was used for data evaluation. *p* < 0.05 was considered significant

### Dysphagia

Dysphagia measured with three different instruments displayed similar outcomes. There was a significant reduction of dysphagia for both groups. The data derived with the Ogilvie score and with the dysphagia module (QLQ-OG25) are suggestive of better dysphagia relief for the fcSEMS at 3 months; but these differences did not reach statistical significance (0.081 and 0.067, respectively) (Table 3). Similarly, the differences in the improvement of dysphagia measured with the Watson score seemed in favor of the fcSEMS stent, but this was not statistically substantiated (*p* = 0.107). There were no differences in any of the scores at the 1 week and 4 week observation points (Table 3).

**Table 3:** Dysphagia measured with three different scales.

	scSEMS	fcSEMS	*p* value
Watson score			
Prestent dysphagia	34.0 (0.0–45.0) *n* = 45	36.7 (5.5–45.0) *n* = 44	0.138
1 week	30.0 (0.0–44.0) *n* = 33	29.5 (0.0–45.0) *n* = 30	0.731
1 month	22.5 (4.5–40.0) *n* = 27	21.7 (0.0–45.0) *n* = 20	0.897
3 months	19.0 (0.0–44.5) *n* = 12	4.0 (0.0–40.5) *n* = 9	0.125
Ogilvie			
Prestent dysphagia	2.0 (2.0–4.0) *n* = 45	3.0 (2.0–4.0) *n* = 45	0.565
1 week	1.5 (0.0–3.0) *n* = 33	2.0 (0.0–4.0) *n* = 32	0.190
1 month	1.0 (1.0–3.0) *n* = 27	1.0 (0.0–3.0) *n* = 22	0.483
3 months	1.0 (0.0–4.0) *n* = 12	0.0 (0.0–3.0) *n* = 9	0.081
QLQ-OG25 Dysphagia module			
Prestent dysphagia	66.7 (11.1–100.0) *n* = 43	77.8 (11.1–100.0) *n* = 43	0.479
1 week	33.3 (0.0–100.0) *n* = 33	44.4 (0.0–100.0) *n* = 30	0.766
1 month	33.3 (0.0–77.8) *n* = 27	44.4 (0.0–77.8) *n* = 21	0.842
3 months	22.2 (0.0–100.0) *n* = 12	0.0 (0.0–44.4) *n* = 8	0.067

The two-tailed Mann–Whitney *U* test was used for data evaluation. *p* < 0.05 was considered significant. Results are presented as median, range, and *n* = numbers of patients per group

### QoL

Overall QoL was measured with EORTC QLQ-C30. Both groups expectedly reported a similarly low global QoL score at randomization. No significant difference in overall QoL between the two stent designs was found during follow-up (Table 4).

**Table 4:** Overall Quality of life measured with QLQ-C30.

	scSEMS	fcSEMS	*p* value
Prestent QoL (QLQ-C30) median (min–max)	41.7 (0.0–91.7)	33.3 (0.00–83.3)	0.212
1 week median (min–max)	41.7 (0.0–83.3)	25.0 (0.0–66.7)	0.107
1 month median (min–max)	41.7 (0.0–100.0)	33.3 (0.0–66.7)	0.260
3 months median (min–max)	62.5 (8.3–83.3)	41.7 (0.0–75.0)	0.142

Two-tailed Mann–Whitney *U* test was used for data evaluation. *p* < 0.05 was considered significant. Higher value means better perceived QoL. Quite low values were found initially. A random sample of Swedish adults in the same age group has a mean value of 77.5 (SD 22.0)

### Survival

Kaplan–Meier analysis with the log-rank test showed no significant differences in survival between the groups (*p* = 0.53).

### Re-interventions

There were 15 (scSEMS) vs. 8 (fcSEMS) interventions in total over the studied 3 months, where it had been deemed necessary to perform a new endoscopy due to the patient’s complaints or symptoms. Again a tendency toward a lesser need for re-interventions was seen in the fcSEMS group (*p* = 0.083) (Table 2). The re-interventions in the scSEMS group included the following: 1 patient with a stent dislocation that needed repositioning; 2 patients who needed a new stent due to complete dislocation; 5 patients with well-placed stents and no endoscopy findings that could explain the patient’s complaints; 2 patients with tumor in-growth and 5 patients with food impaction. Corresponding numbers in the fcSEMS group were: 3; 3; 2; 0, and 0, respectively.

### Complications

There was only one serious complication. A patient in the scSEMS group experienced a perforation at stent insertion on day 0 and received emergency surgery. Other complications that were minor included bleeding, reflux, and pain with no differences between the groups.

## Discussion

For patients with inability to swallow due to an advanced malignancy of the esophagus and GEJ, it is of critical importance to treat the dysphagia and alleviate symptoms. From a patient perspective, it is of great importance that such treatment is safe and effective, and has few side effects, thus minimizing additional interventions. Stent placement has the benefits of providing a well-documented and rapid onset of the effect and is often accessible close to the patient since it can be performed at most local endoscopy units. One challenge is of course to choose an optimal stent type that carries a minimal risk for stent migration and other complications.

In this prospective, randomized study, we aimed to establish whether modifications of an fcSEMS aimed at maintaining its positioning could be sufficient to reduce its tendency to migrate, a phenomenon that often causes symptoms and necessitates re-interventions. Given that earlier studies have consistently shown that fcSEMS are more likely to dislocate than scSEMS [11–15], we hypothesized that this would still be the case despite of the novel design modifications undertaken to prevent such migration. We found however no difference between the two stent models with regard to the actual distance of migration or the migration frequency. It would appear, then, that the novel design modifications of the fcSEMS, i.e., a flare in both ends with a modified shape and the lining located at the interior side leaving bare mesh on the exterior stent surface, are indeed effective in reducing fully covered stents’ tendency to migrate. Interestingly, this was in spite of the somewhat lower radial force the Wallflex^®^ fcSEMS exhibits compared to the conventional Ultraflex^®^ scSEMS used here. The finding that the need for endoscopic re-interventions was, if anything, lower in the fcSEMS group, further supports the comparable patency of the fcSEMS vs. the scSEMS. Our findings are also consistent with a previous study on malignant strictures in which the safety and efficacy of the fully covered Wallflex^®^ stent were investigated [27]. In that study, however, individual-based effects before and after stent placement were compared with historical data, and no randomized control arm was included [27]. Regardless of these limitations, the inserting of a fcSEMS reduced the risk of recurrent dysphagia due to dislocation, and improved dysphagia scores similar to our findings (Table 3) as well as to earlier descriptive studies on scSEMS [11, 16, 17].

In contrast to earlier investigations [8, 9, 11–13, 15–17, 27–30], we applied a unique but simple method to objectively evaluate stent migration. We used chest X-rays, which allows objective measures of the actual stent position to be taken directly, permitting quantification of any possible changes during the follow-up. It may be argued that small movements of the SEMS may be of marginal clinical importance, and that perceived symptoms and re-interventions rather than direct objective measures of migration distance should therefore be the preferred primary outcome variable. Although it is of vital importance to take into consideration subjective symptoms in clinical palliative care, it is well known that patients with advanced cancer of the esophagus and GEJ do experience a variety of symptoms in general, which may change from day to day [31–33]. As it was evident in the present study, these symptoms are not necessarily always related to stent migration or dislocation. Thus, if re-interventions decided solely by symptoms are used as the main study outcome variable and as a proxy for migration, it is possible that study patients risk having to undergo some unnecessary invasive endoscopic investigations. Stents as such can also cause symptoms even when they remain in the correct position [14], and conversely movements of the stents may well be missed unless the patient experiences symptom aggravation. Using direct X-ray aided measurements in the studies of stent migration, the location of the stent may be determined more objectively and without burdening the patients with additional invasive procedures unless needed.

In addition to investigating stent migration and allowing for comparisons to be made with earlier studies in which symptoms were used as the main outcome variables, we evaluated dysphagia, QoL, re-interventions, technical failures, and survival as secondary variables. As expected, individual dysphagia scores were improved in response to SEMS placement at all time points. We even noted a tendency toward less dysphagia in the fcSEMS group at 3 months as assessed with the Ogilvie scale and the QLQ-OG25 scale (Table 3). This may suggest that the fcSEMS could be advantageous in providing dysphagia relief in patients with a longer life expectancy. It is difficult to firmly interpret such a potential effect; a limitation of this study is of course that the number of remaining evaluable patients was notably very small in both groups at 3 months, due to the aggressive nature of the cancer disease. In addition, the study was powered with regard to the primary variable, and at a group level, no significant difference between the specific stent designs was detected at any time point with regard to reported dysphagia (Table 4). This latter result notwithstanding, our results motivate a future clinical trial comparing these stent designs powered for measuring dysphagia as the primary outcome variable.

Concerning overall QoL, the scores are concordant to what has previously been reported for corresponding patient groups [3, 34]. We noted that QoL in fact seemed to be unchanged or slightly reduced the first week after SEMS treatment (Table 4). This observation is consistent with earlier studies by Shensi et al. and Madhusudhan et al. [35, 36] suggesting that a stent may cause some initial symptoms that can be negatively perceived such as reflux and pain [14]. Over time, there was an improvement in overall QoL at three months for both stent types, but with no differences between the groups.

In conclusion, this randomized controlled trial shows that the fcSEMS used in this study does not migrate more compared to the conventional scSEMS and indicates that it is at least similar to the conventional scSEMS with regard to migration frequency and distance. Whether newer fcSEMS may have an advantage in providing better dysphagia relief in patients with longer survival expectancy needs to be established.
